# Similarities and differences in structure, expression, and functions of VLDLR and ApoER2

**DOI:** 10.1186/1750-1326-6-30

**Published:** 2011-05-09

**Authors:** Sunil S Reddy, Teal E Connor, Edwin J Weeber, William Rebeck

**Affiliations:** 1 Department of Biology; Georgetown University, 37th and O Streets, NW, Washington, DC, 20057, USA; 2 Department of Neuroscience; Georgetown University Medical Center, 3970 Reservoir Rd, NW, Washington, DC, 20007, USA; 3 Department of Molecular Pharmacology & Physiology; University of South Florida, 4001 E. Fletcher Ave., Tampa, FL, 33612-4742, USA

## Abstract

Very Low Density Lipoprotein Receptor (VLDLR) and Apolipoprotein E Receptor 2 (ApoER2) are important receptors in the brain for mediating the signaling effects of the extracellular matrix protein Reelin, affecting neuronal function in development and in the adult brain. VLDLR and ApoER2 are members of the low density lipoprotein family, which also mediates the effects of numerous other extracellular ligands, including apolipoprotein E. Although VLDLR and ApoER2 are highly homologous, they differ in a number of ways, including structural differences, expression patterns, alternative splicing, and binding of extracellular and intracellular proteins. This review aims to summarize important aspects of VLDLR and ApoER2 that may account for interesting recent findings that highlight the unique functions of each receptor.

## Introduction

Very Low Density Lipoprotein Receptor (VLDLR) and Apolipoprotein E Receptor 2 (ApoER2; also known as LRP8) are members of the LDL receptor family, a group associated with cellular cholesterol homeostasis [1]. LDL receptor family members are type I transmembrane receptors and have five highly conserved structural domains: an extracellular N-terminal ligand-binding domain with cysteine-rich repeats, an epidermal growth factor (EGF) domain, an O-linked sugar domain, a single transmembrane sequence, and a cytoplasmic domain containing an NPxY motif. The overall sequence homology between VLDLR and ApoER2 is around 50% [2].

VLDLR and ApoER2 are primarily recognized for their role in neural development through Reelin signaling, a process that is responsible for proper positioning of newly generated neurons leading to the inside-out formation of the six-layered neocortex [3]. Reelin is a glycoprotein secreted by Cajal-Retzius cells that signals neurons to migrate radially past earlier-generated cells to arrive at the appropriate lamina [4]. Reelin signaling is also important for alignment of pyramidal neurons in the hippocampus and Purkinje cells of the cerebellum [3]. In this pathway, VLDLR and ApoER2 are cell surface receptors for Reelin [5,6]. Interaction with Reelin induces VLDLR and ApoER2 to bind the adaptor protein Dab1 at an NPxY motif on their cytoplasmic tails [7,8]. Binding of Dab1 subsequently leads to activation of Src family tyrosine kinases (SFKs) and other kinases that phosphorylate the adaptor protein at its tyrosine residues [9]. VLDLR and ApoER2 are the exclusive Reelin signaling receptors, and, consistent with their high extracellular sequence homology, bind Reelin with similar affinities [10]. Mutations or deletions of the *Reelin *gene, the *Dab1 *gene, or the genes for both *VLDLR *and *ApoER2 *result in improper neuronal migration, cortical laminating defects, and cerebellar hypoplasia [5,7,11,12].

Secreted Reelin can form complexes, including homodimers, which allows association and clustering of multiple receptors simultaneously [9,13]. This receptor clustering is necessary for the phosphorylation of Dab1 and its subsequent interaction with kinases [9]. Ligand binding to VLDLR and ApoER2 also initiates clathrin-dependent receptor endocytosis, which likely modulates further ligand signaling [14]. Dab1 phosphorylation following Reelin activation of apoE receptors leads to Dab1 degradation by proteosomes [15]. Thus, there are several mechanisms for regulating the Reelin signaling mechanisms.

Studies of the developing CNS suggest that VLDLR and ApoER2 serve overlapping functions in controlling neuronal migration. However, several recent studies have demonstrated clear differences in VLDLR and ApoER2 splicing, localization, interactors, and trafficking. The differences in receptor function during development also translate into differences in adult brain function. Both VLDLR and ApoER2 are receptors for apolipoprotein E (apoE), the leading genetic risk factor for Alzheimer's disease (AD) [16], and these receptors may play different roles in modulating the risk associated with apoE. Here, we explore VLDLR, ApoER2, and importantly, the distinctions between them.

### Comparison of VLDLR and ApoER2 proteins

#### Structure

**Figure 1:**
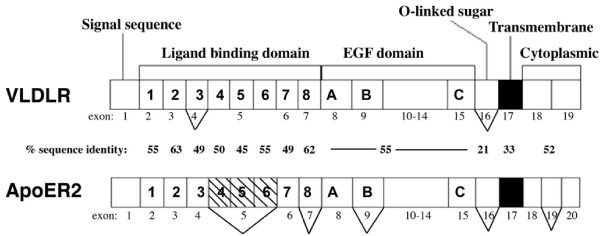
**VLDLR and ApoER2 receptors**. Specific domains are defined by vertical lines, alternative splice forms are shown with protruding "v"s, exon numbers are noted, and amino acid %-sequence identities of individual domains are indicated.

Full length human *VLDLR *mRNA encodes a protein of 873 amino acids ([17]; NCBI Accession NM_003383.3). VLDLR has eight cysteine-rich ligand-binding repeats (LBRs) of approximately 40 amino acids each, an EGF region with three cysteine rich repeats, a 46 amino acid O-linked glycosylation domain with numerous threonine and serine residues, a 22 amino acid transmembrane domain, and a 54 amino acid cytoplasmic domain containing the NPxY motif (Figure 1) [18]. The first 27 amino acids of the N-terminal are hydrophobic and serve as the signal peptide. A glycine residue is believed to constitute the N-terminus of the mature receptor. There is 97% amino acid identity between VLDLR in mouse and human [17].

The full length human *ApoER2 *mRNA encodes a protein of 870 amino acid residues (NCBI Accession NM_004631.3). ApoER2 has the same overall domain structure as VLDLR, with several important differences. ApoER2 contains a ligand-binding domain of eight LBRs (only five LBRS are ever observed in the cDNA due to splicing out of LBRs 4-6; see Figure 1), an EGF domain with three cysteine-rich repeats, an O-linked glycosylation domain of 89 amino acids, a transmembrane domain of 24 amino acids, and a cytoplasmic domain of 115 amino acids (Figure 1) [2]. The signal peptide consists of approximately 40 amino acids. Homology between ApoER2 in mouse and human is approximately 90%. In humans, VLDLR and ApoER2 share a roughly 50% primary sequence homology, with the O-linked glycosylation domain (21%) and transmembrane domain (35%) being the least identical [2].

Thus, the gene sequences of *VLDLR *and *ApoER2 *differ most dramatically in their O-linked glycosylation domains; this domain in ApoER2 is more than twice the size of that in VLDLR.

#### Distribution (Temporal and Spatial)

*VLDLR *mRNA transcripts are most abundant in brain with high expression in the heart and skeletal muscle as well (Table 1). These tissues have relatively high levels of free lipids, supporting a role of VLDLR in metabolism of cholesterol and fatty acids [18]. Within the adult brain, *VLDLR *mRNA is found in nearly all regions, with highest expression in the cortex and cerebellum (Table 2). Within these regions, VLDLR is found on microglia and region-specific pyramidal neurons [19], as well as in neuroblasts, matrix cells, Cajal-Retzius cells, glioblasts, astrocytes, and oligodendrocytes [18,20] (Table 3). Subcellularly, VLDLR is in non-lipid raft fractions of cell membranes [14].

**Table 1:** Expression of VLDLR and ApoER2 in mouse organs. +/-, +, and ++ represent increasing levels of expression [2,18].

	Brain	Heart	Testis	Ovary	Kidney	Muscle	Placenta	Lung	Adipose Tissue	Small Intestine	Liver
VLDLR	++	++	+	+	++	++	-	+	+	+/-	+/-

ApoER2	++	-	++	+/-	-	-	+	-	-	-	-

**Table 2:** Regional expression of VLDLR and ApoER2 within adult mouse brain.

Brain:	TH	CX	ST	HI	HY	CB	BR	OB	SE
VLDLR	+	+	+	+	+	+	+	-	+

ApoER2	-	+	-	+	-	+	-	+	-

TH = Thalamus, CX = Cerebral Cortex, ST = Striatum, HI = Hippocampus, HY = Hypothalamus, CB = Cerebellum, BR = Brainstem, OB = Olfactory bulb, SE = Septum [2,18].

**Table 3:** Cellular expression of VLDLR and ApoER2 within mouse brain [2,18,21].

	Glia	Cortical Plate Neurons	Hippocampal Pyramidal Neurons	Cajal-Retzius cells	Olfactory Mitral Cells	Cerebellar Purkinje Cells
VLDLR	+	++	+	++	-	+

ApoER2	-	++	+	-	++	++

*ApoER2 *mRNA is present exclusively in CNS, placenta, and testis [2] (Table 1). Immunohistochemical analysis reveals ApoER2 expression in the hippocampus, cerebellum, mitral cell layer of the olfactory bulb, neocortex (cell bodies and dendrites), and cortical neurons [21,22] (Table 2). In the hippocampus, ApoER2 is found in granule cells of the dentate gyrus and pyramidal cells of all CA subfields [21]. In the cerebellum, ApoER2 is found densely in Purkinje cells [2,21] (Table 3). Subcellularly, ApoER2 is differentially sorted into lipid raft fractions of cell membranes [14].

*VLDLR *and *ApoER2 *also have different patterns of tissue expression over development (Table 4). At E12 of development both *VLDLR *and *ApoER2 *are expressed at low levels in the preplate. From E12.5-E13.5, *ApoER2 *is expressed in the inner and middle locations of the cortex; and at E15, *ApoER2 *is in the olfactory bulb and in medial cortical fields. At E15, *VLDLR *is seen in the hippocampal anlage-medial cortex, paleocortex, subplate of the upper cortical plate, and weakly in the neocortical plate and Cajal-Retzius cells. From E16.5-E17 *ApoER2 *expression is in the outermost cortex as well as deeper cerebellar layers, and at E18, *ApoER2 *is expressed in the same regions as VLDLR was at E15, with the exception of Cajal-Retzius cells. *VLDLR *at E18 maintains the same as its E15 expression pattern but with stronger expression in Cajal-Retzius cells, and signal in Purkinje cells. Postnatally, from P0-P6, both *VLDLR *and *ApoER2 *are expressed in the subplate and pyramidal cells of layers III and V; *VLDLR *is additionally in Cajal-Retzius and Purkinje cells postnatally [22,23]. Thus, *VLDLR *is expressed earlier in development and is more highly expressed in Cajal-Retzius cells than *ApoER2*.

**Table 4:** Temporal and spatial expression of *VLDLR *and *ApoER2 *within developing embryonic mouse brain [22,23].

Developmental day	E12	E12.5-13.5	E15-E15.5	E16.5-E17	E18	P0-P6
VLDLR	Low expression in preplate	Intermediate zone and cortical plate	Developing hippocampus; palaeocortex; neocortical plate; Cajal-Retzius cells; subplate; deep layers of cerebellum	All areas as E15	All areas as E15, plus: Stronger expression in Cajal-Retzius cells; Purkinje cells	Subplate and pyramidal cells of layers III and V; Cajal-Retzius cells; Purkinje cells

ApoER2	Low expression in preplate	Inner and middle locations of cortex	Medial cortical fields; olfactory bulb; ubiquitously in cerebellum	Outermost cortex; deeper cerebellar layers	Developing hippocampus; palaeocortex; neocortical plate, subplate	Subplate and pyramidal cells of layers III and V

#### Splicing

Both *VLDLR *and *ApoER2 *undergo alternative splicing to produce several transcript variants. Four transcripts of *VLDLR *(I-IV) have been defined [20]. *VLDLR*-I, lacks none of the exons, *VLDLR*-II lacks exon 16 encoding the O-linked sugar domain, *VLDLR*-III lacks exon 4 encoding the third ligand-binding repeat, and *VLDLR*-IV lacks both exons 4 and 16. In adult mouse brain, over half (75%) of transcripts lack exon 16 and the next most common form lacks both exons 4 and 16. Thus, most of the VLDLR present in the brain lacks the O-linked sugar domain and thus would not be expected to be glycosylated. Astrocytes exhibit less splicing of exon 4 and exon 16 than neurons [20]. *VLDLR *containing exon 16 is increased in normal aging and Alzheimer's disease [24].

*ApoER2 *displays a more complex splicing pattern. Exon 5 (Figure 1), containing ligand-binding repeats 4-6, is spliced out of all transcripts [21,25]. The eighth LBR is spliced out during very early stages of development (E12 in mouse). Some transcripts in brain and all transcripts in placenta contain a 13 amino acid insert containing a furin cleavage site in place of ligand binding repeat 8 (exon 7) [25]. Exon 9, encoding an EGF repeat, and exon 16, containing the O-linked sugar domain, can be alternately spliced out [21], resulting in a smaller, differentially glycosylated precursor form found in the ER [26]. Splicing of the ligand-binding and EGF repeats alters binding affinity to ligands, suggesting the different transcripts may function in fine-tuning receptor-ligand interactions [27]. Finally, *ApoER2 *contains an alternatively spliced exon (19) that encodes a 59 amino acid domain in the cytoplasmic tail [2,21,25]. The exon 19 domain is proline-rich and ends 10 amino acids before the C-terminus of ApoER2. The presence of this domain promotes interactions of ApoER2 with the c-jun N-terminal kinase (JNK) interacting proteins, JIP1 and JIP2, and the synaptic protein, PSD-95 [28,29] (see below).

Thus, differential splicing of ApoER2 and VLDLR produce proteins with different numbers of LBRs and very different cytoplasmic domains.

#### Interacting proteins

**Figure 2:**
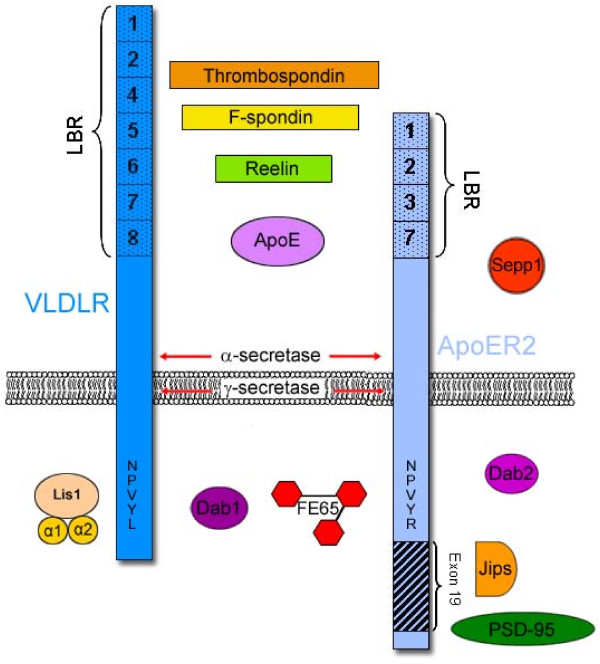
**VLDLR and ApoER2 interacting proteins**. Some of the extracellular and intracellular proteins that interact with VLDLR and ApoER2 are indicated.

VLDLR and ApoER2 interact with a similar, but not identical, number of proteins [30]. Extracellularly, these receptors mediate the effects of specific ligands. Intracellularly, protein interactions alter receptor trafficking and processing [31]. In addition, proteolytic enzymes act on the receptors to generate N- and C-terminal fragments [32,33]. We summarize some of these interacting proteins below (see also Table 5 and Figure 2).

**Table 5:** Interactions of various molecules with VLDLR and ApoER2.

VLDLR		Ectodomain Interactor		ApoER2
unknown	**+**	Reelin	**+**	LBR1

LBR	**+**	Thrombospondin F-spondin	**+**	LBR

LBR 5-8	**+**	ApoE	**+**	LBR 1-3, 7

	-	selenoprotein	**+**	unknown

				

**VLDLR**		**Transmembrane Interactor**		**ApoER2**

Releases intracellular domains	**+**	γ-secretase	**+**	Releases intracellular domains

Releases extracellular domains	**+**	α-secretase	**+**	Releases extracellular domains

				

**VLDLR**		**Cytoplasmic Interactor**		**ApoER2**

PTB domain binds 14-residue peptide around the NPxY	**+**	Dab1	**+**	PTB domain binds 14-residue peptide around the NPxY

	-	Dab2	**+**	PTB domain binds NPxY of ApoER2

NPxY followed by leucine	**+**	Pafah1b2 & Pafah1b3	-	NPxY followed by arginine

	-	JIP-1, JIP-2	**+**	Exon 19

	-	PSD-95	**+**	Exon 19

NPxY	**+**	FE65	**+**	NPxY

##### Extracellular

Reelin is a large extracellular matrix protein (~400 kDa) expressed by Cajal-Retzius cells during embryonic development and by interneurons in the postnatal brain [3,6]. Reelin can bind to both VLDLR and ApoER2 in the absence of a co-receptor [6,34], although a co-receptor may be needed for efficient binding [35]. Reelin binds to the first ligand binding repeat of VLDLR and ApoER2; this interaction depends on a specific lysine residue in Reelin (amino acid 2467) [36]. Reelin is cleaved at numerous sites to generate a series of fragments [37,38]. Reelin fragments bind equally to both VLDLR and ApoER2, but native full-length Reelin binds with higher affinity to ApoER2 (LBRs-12378) than to VLDLR [38]. Reelin fragments with cleaved C-terminal regions bind VLDLR and ApoER2 with a significantly higher affinity than full-length Reelin [27].

Apolipoprotein E (apoE) is a component of lipoproteins involved in phospholipid and cholesterol homeostasis [39]. VLDLR binds and internalizes all human isoforms of apoE (E2, E3, E4) with high affinity, including lipid-free forms, and it does so through LBRs 5-8 [39]. The ApoER2 LBRs binds apoE containing β-VLDL with high affinity, specifically the ApoER2 splice variants that lack LBR's 4-6 and LBR 8 [25]. ApoE bound to receptors may be internalized and degraded, recycled to the cell surface, or remain intracellular for extended periods [40]. ApoE leads to the cleavage of both VLDLR and ApoER2 to generate secreted forms of these receptors [33].

Thrombospondin and F-spondin are extracellular matrix proteins. Thrombospondins are a family of trimeric or pentameric proteins that are involved in cell-cell and cell-matrix communication, and affect processes such as neurite outgrowth, neuronal migration, and synaptogenesis [41,42]. Thrombospondin-1 binds to the ligand binding repeats of VLDLR and ApoER2, and competes with Reelin binding [43]. Like Reelin, Thrombospondin can induce Dab1 phosphorylation although not the subsequent Dab1 degradation [43]. F-spondin is a secreted molecule whose C-terminal domain is similar to repeats found in Thrombospondin; its N-terminal domain is homologous to a domain in Reelin that promotes dimerization [44]. The thrombospondin domain of F-spondin interacts with the LBR domain of ApoER2 [45] and may bind to both VLDLR and ApoER2 in the rostral migratory stream [46]. Like Reelin and Thrombospondin, F-spondin can induce activation of Dab1 [47].

Selenium is a ligand for ApoER2 through its transporter, Selenoprotein P (Sepp1). Sepp1 transports selenium from the liver to other target tissues such as the testis and brain [48]. ApoER2 knockout mice have phenotypic similarities to Sepp1 knockout mice such as lower selenium levels in areas where ApoER2 is normally expressed (the brain and the testis) [49]. When fed a selenium deficient diet, ApoER2 knockouts had neurological problems, progressive neurodegeneration, and shortened survival, similar to Sepp1 knockouts on the same diet [49,50]. ApoER2 mediates the endocytosis of Sepp1 [48].

##### Membrane Spanning Domain

Secretases promote cleavage of VLDLR and ApoER2 [32], and these proteolytic events can be induced by ligand binding [33]. A soluble extracellular fragment is generated by metalloproteinase activity; cleavage of ApoER2 occurs at small levels even when unstimulated, but after Reelin stimulation cleavage significantly increases [33]. γ-secretase cleaves in the intramembrane regions to generate soluble intracellular domains [32]. This cleavage was not observed with a monovalent ligand, the receptor-associated protein RAP, suggesting the importance of multivalent, clustering-ligands in this process [9]. ApoE leads to release of extracellular domains and increased accumulation of C-terminal fragments of both VLDLR and ApoER2, but with less cleavage apparent for VLDLR than for ApoER2 [33].

Proteolysis of VLDLR and ApoER2 can also be promoted by extracellular interactions of the receptors with the proprotein convertase PCSK9 [51]. PCSK9 was first associated with degradation of the LDL receptor [52], and is regulated by SREBP [53]. In addition, proteosomal degradation of VLDLR and ApoER2 can be induced by an E3 ubiquitin ligase (IDOL, Inducible Degrader of the LDL Receptor) [54]. IDOL is regulated under the LXR system [54], which is important for cellular cholesterol homeostasis. Thus, VLDLR and ApoER2 levels can be regulated in sterol-responsive ways, similar to the process for the LDL receptor.

##### Intracellular

Disabled-1 binds to VLDLR and ApoER2 intracellularly at NPxY motifs through its phosphotyrosine-binding (PTB) domain [8]. The PTB domain of Dab1 contains a hydrophobic groove and this structure creates the preference for the ApoER2 C-terminal helix [55]. Dab1 also binds Src and Fyn kinases, which are important in the downstream organizational effects of Reelin signaling [56]. The PTB structure has steric interference with phosphorylated tyrosines, explaining the preference for the NPxY motifs containing unphosphorylated tyrosines [55]. The presence of extracellular ligands such as Reelin increases the interaction of ApoER2 with Dab1 [57,58]. Reelin-binding of VLDLR and ApoER2 result in similar and robust levels of Dab1 phosphorylation, and receptor-clustering mimics this effect [26]. Dab1 promotes surface localization of ApoER2 and its subsequent cleavage by α-secretase [58].

Disabled-2 (Dab2), like its homolog Dab1, is a cytosolic adaptor protein. Dab2 is found at various locations in the embryonic mouse CNS throughout development, including the developing hindbrain, floor plate of the neural tube, choroid plexus, subcommissural organ, and pineal gland [59]. Like Dab1, Dab2 is thought to play a role in organization and cell positioning by signaling through LDL receptor family members [60], but it does not compensate for loss of Reelin signaling. Dab2 has been shown to regulate the MAPK pathway as well as Src activity [61]. Dab2 is also necessary for the internalization of ApoER2; the PTB domain of Dab2 interacts with the NPxY motif of ApoER2 and leads to internalization independent of exon 19 [62]. Dab2 does not bind VLDLR [60].

The Pafah1b complex of proteins consists of Lis1 (encoded by the *Pafah1b1 *gene) and two catalytic α subunits (encoded by *Pafah1b2 *and *Pafah1b3*) [63]. Heterozygous mutations of *Pafah1b1 *cause lissencephaly and a reduced number of cortical gyri, similar to the *reeler *phenotype [64]. In vitro, the α-subunits bind VLDLR at its NPVYL domain but not ApoER2, which has an NPVYR sequence [65]. Mouse models also support the conclusion that Pafah1b is functionally connected to VLDLR but not ApoER2 [65].

JIP-1 and JIP-2 act as molecular scaffolds in the JNK-signaling pathway [66]. JIP-1 and JIP-2 were identified in a yeast two-hybrid assay as interactors with the alternatively-spliced exon 19 of ApoER2, and thus do not bind VLDLR, which lacks this domain [31]. The PID domain, rather than the SH3 domain, of JIP-2 was found to be responsible for the interaction with exon 19 [28]. The binding of JIPs and Dab1 to ApoER2 suggests that ApoER2 forms a scaffold for various interactors at the cell surface [28].

PSD-95 is another adaptor protein that interacts preferentially with exon 19 of ApoER2 [29,31,67]. The first PDZ (PDZ1) domain of PSD-95 is responsible for the interaction with the intracellular exon 19 of ApoER2 [67]. PDZ2 of PSD-95 binds the NR2 subunit of NMDA receptors, possibly responsible for the complexes of ApoER2, NR2A, and PSD-95 formed in post-synaptic densities [29].

FE65 has three functional domains: two PTB domains, and one WW domain [68]. FE65 binds to NPxY sequences in members of the LDL receptor family via its PTB1 domain [31,69]. FE65 interacts with ApoER2 [31,69] and VLDLR (GWR, unpublished data). The other domains of FE65 are important for linking apoE receptors to other proteins, such as the amyloid precursor protein via PTB2 [70], and to the cytoskeleton via the WW domain, affecting cell movement [71]. Knock-out of *FE65 *family members results in lissencephaly [72], as for several of the other proteins that interact with apoE receptors (see above).

### Unique functions of VLDLR and ApoER2

One of the main differences between VLDLR and ApoER2 is that ApoER2 is restricted to the brain, testis, and placenta whereas VLDLR is expressed in tissues throughout the body. Both receptors are widely expressed in the brain, both during development and in the adult. Knockout mouse models of only *ApoER2 *or only *VLDLR *do not show the dramatic neuronal migration defects seen in the mice lacking both receptors, a phenocopy of *Reelin *and *Dab1 *knockouts [5]; this observation led to the hypothesis that the receptors have overlapping functions in the brain. However, several recent studies have begun to define distinct functions of VLDLR and ApoER2; we will discuss these distinctions in this section.

#### Contributions to neuronal migration

Reelin interacts extracellularly with VLDLR and ApoER2, promoting Dab1 phosphorylation intracellularly, and resulting in downstream effects that regulate proper migration of neurons during development [6,34,73]. As mentioned, double *VLDLR*/*ApoER2 *knock-outs show the full *reeler *phenotype, similar to animals lacking *Reelin *or *Dab1 *[74]. However, analyses of the single knock-out mouse models provide insight into different functions of ApoER2 and VLDLR. Both *ApoER2 *and *VLDLR *knock-outs have smaller, less foliated cerebella, less cortical lamination, and a splitting of the CA1 layer of the hippocampus [5]. However, the cortical and hippocampal defects were more pronounced in the *ApoER2 *knock-outs, and the cerebellar defect was more pronounced in the *VLDLR *knock-outs [5].

In addition to these deficits, the *VLDLR *knock-out neurons demonstrated greater invasion of migrating cortical neurons into the marginal zone [5,23]. Furthermore, fate mapping demonstrated that in *ApoER2 *knock-out mice, the late-born neurons fail to migrate properly in the cortex [23]. In *VLDLR *knockouts, the early-born cells ended up in an organized layer of the inner cortex (similar to wild-type animals), suggesting that VLDLR does not have a substantial effect on the limited migration of early-born neurons [23]. VLDLR is primarily expressed in the intermediate zone and the cortical plate adjacent to Reelin-expressing cells in the marginal zone [5]. These observations support a hypothesis that VLDLR mediates a neuronal "stop signal" [23]. In WT mice, ApoER2 receptors are most strongly expressed at E16.5 and E18.5, the stage when late-born neurons reach the superficial layers of the outer cortex. In the *ApoER2 *knock-out, the early-generated layers formed with minor deficits but late-generated layers were highly disrupted [23]. Antibodies against molecules of the radial glial scaffold revealed no abnormal arrangement of the radial glial cells. In *ApoER2 *knock-outs there is strong disruption of the radial alignment of the cortical neurons; this phenotype is not seen in the *VLDLR*-deficient cortex in which neurons are distributed radially [5]. Furthermore, neurons in *ApoER2 *knock-outs are packed into horizontal layers while the *VLDLR *knock-outs do not demonstrate discernable cortical lamina [5].

Human mutations in the Reelin gene (*RELN*) gene cause neuronal migration defects, producing alterations in the architecture of the cortex and cerebellum [75]. The structural phenotype of lissencephaly, malformed hippocampus, and cerebellar hypoplasia is associated with a clinical phenotype of hypotonia, ataxia, and severe mental retardation [75]. Humans have also been identified with homozygous deletion of the *VLDLR *gene [76]. The *VLDLR *deletion causes a more mild cerebral gyral simplification, less cerebellar hypoplasia, and unremarkable changes to the hippocampus, compared with the *RELN *mutation [76]. Thus, the observations with mouse knock-out models support the findings in human genetic diseases.

#### Pafah1b effects on neuronal migration via VLDLR

Several of the proteins that interact with VLDLR and ApoER2 have been identified through mouse knock-out models that demonstrate effects on neuronal migration. The lissencephaly phenotype of *Pafah1b1 *heterozygous mutants sparked interest in exploring the relationship between the Pafah1b complex and the Reelin pathway, including the VLDLR and ApoER2 receptors [64]. The *Pafah1b1*^+/-^;*ApoER2*^-/- ^double mutants had severe deficits in the forebrain, including inversion of cortical-lamina and hypercellularity of layer I similar to the *reeler*-like phenotype of an *ApoER2*;*VLDLR *double knock-out. However, *Pafah1b1*^+/-^;*VLDLR*^-/- ^double mutants were mostly normal. The *Pafah1b1*^+/-^;*ApoER2*^-/- ^mutants still underwent Dab1 phosphorylation when stimulated with Reelin (unlike in *reeler *mice), demonstrating that signaling was still intact (via VLDLR) but was not enough for proper cortical layer formation (requiring Lis1). The α-subunits serve as adapter-molecules by bringing Lis1 to VLDLR; one of the subunits (α2) binds to Dab1 [77]. The intact signaling suggests Lis1 is downstream of VLDLR and SRKs [65]. In humans, deletions and duplications of the *PAFAH1B1 *also result in neuronal migration defects, leading to a reduction in the number of cortical gyri [63,78,79]. Like the human *VLDLR *homozygous deletion [76], the *PAFAH1B1 *heterozygous deletion causes a somewhat milder phenotype than *RELN *mutations [75]. Thus, the Pafah1b complex mediates downstream effects of VLDLR on neuronal migration, but is not necessary for the function of ApoER2.

#### Lipid-raft sorting

Lipoprotein receptors are associated with clathrin-coated pits, indicative of a role in ligand endocytosis and degradation. However, ApoER2 is only found in caveolae-rich membrane fractions [26,80], distinguishing it from the non-lipid-raft VLDLR. Caveolae are a type of lipid raft primarily involved in signaling pathways [80]. Chimeric ApoER2 constructs containing only its cytoplasmic tail do not associate into rafts, suggesting it is the transmembrane or ectodomain involved in the sorting [26]. The transmembrane domain of ApoER2 has only 33% sequence identity compared with VLDLR (Figure 1); this low level of homology may be related to differential sorting of ApoER2 into lipid rafts.

Thus, the different localization of ApoER2 and VLDLR may play a role in their different functions. Reelin is endocytosed via clathrin-coated vesicles and degraded by VLDLR much more efficiently than by ApoER2 [14]. In cell culture experiments, VLDLR facilitated degradation of more than 60% of bound Reelin after 12 minutes, and all of Reelin by 24 minutes. With ApoER2, after 24 minutes ~75% of Reelin remained associated with the cells [14]. Disruption of raft structures increased ApoER2-mediated Reelin endocytosis to the rate of VLDLR, indicating that ApoER2's localization in rafts results in reduced endocytosis. Reelin-binding resulted in a lysosomal (rather than proteosomal) degradation of ApoER2 but not of VLDLR, independent of raft-association [14]. However, ApoER2 bound by the non-clustering ligand RAP (receptor-associated protein) did not result in down regulation, suggesting that degradation is mediated by receptor clustering [14]. Thus, ApoER2 may be more important for the mediation of Reelin signaling in lipid rafts, and VLDLR may be more important for the intracellular degradation of Reelin.

#### Neuronal signaling

Activation of ApoE receptors directly promotes signal cascades involving the intracellular adaptor protein Dab1, increased activation of SFKs, and decreased activation of JNK [81]. Indirect activation of signaling involving calcium-mediated signal transduction pathways occurs through modification and subsequent increase in NMDAR conductance, which in turn can activate the extracellular signal-related kinases 1/2 (ERK1/2) [29,82]. It is unclear what the specific contributions are for each ApoE receptor or ligand; however, the use of the ligand Reelin has allowed for the identification of at least three specific pathways associated with ApoER2 and VLDLR activation. First, acute Reelin application to hippocampal slices results in NMDA receptor phosphorylation of both NR2A and NR2B subunits that is dependent on Src activation [28,29,67]. Second, chronic Reelin treatment of cultured hippocampal neurons shows a significant decrease in the number of silent synapses by increasing AMPA receptor insertion at synaptic terminals that is dependent on PI3K signaling [83]. Third, ApoE receptors affect the activation state on JNK through unstudied mechanisms [28,29,67]. JNK-interacting proteins JIP-1 and JIP-2 interact with exon 19 of ApoER2 [28,29,67], potentially affecting JNK signaling in many processes such as cell proliferation, morphogenesis and apoptosis [66]. In general, the interactions with exon 19 and consequent downstream signaling effects may explain some of the differences observed between ApoER2 and VLDLR. PSD-95 could act as a link between ApoER2 and NMDA receptors since the PDZ1 domain of PSD-95 binds ApoER2 and the PDZ2 domain of PSD-95 binds NMDA receptor subunits [67]. Thus, the formation of multimeric complexes of signaling proteins with ApoER2 may mediate specific signal transduction mechanisms.

Analysis of *ApoER2 *and *VLDLR *knockout mice demonstrate that there are differential contributions of VLDLR and ApoER2 to hippocampal synaptic plasticity. Mice deficient for ApoER2 have a greater deficit in hippocampal area CA1 long-term potentiation (LTP) compared to VLDLR deficient mice, in particular when a theta-burst high frequency stimulation protocol is used [82]. The addition of Reelin can enhance LTP, but this requires the presence of both receptors [29,82]. This dual receptor dependency may reflect the necessity for the activation of signal transduction pathways from both receptors (described above) or the need for ApoER2/VLDLR clustering during Reelin binding. Nevertheless, current experimental evidence suggests that ApoER2 plays a signaling role independent of VLDLR. For example, the *in vitro *increase in LTP in the presence of Reelin requires interaction between ApoER2 and NMDA receptors through exon 19 of ApoER2 [28,29,67]. ApoER2 knockout mice or mice lacking exon 19 of ApoER2 show no Reelin-dependent changes in NMDARs or increase in LTP induction.

## Conclusions

VLDLR and ApoER2 have homologous extracellular and intracellular domains and each is important for Reelin-mediated neuronal migration that depends on Dab1. Both undergo a series of proteolytic events that result in soluble forms of the receptors and intracellular domains, and that regulate active receptor levels. However, VLDLR and ApoER2 also interact with different ligands that define separate roles for these receptors in various signaling pathways. VLDLR has a broader tissue distribution and interacts specifically with the Pafah1b complex. ApoER2 is found in lipid rafts and interacts with PSD-95 and JIPs through a unique alternatively spliced sequence. These interactions affect signaling pathways regulated by intracellular calcium levels and JNK. As we continue to examine these receptors, we will better define their unique roles in neuronal migration and synaptic signaling.

## Competing interests

The authors declare that they have no competing interests.

## Authors' contributions

SSR did the literature review on ApoER2 and VLDLR comparisons, and TEC compiled and analyzed data. EJW and GWR suggested topics and edited the manuscript. All authors read and approved the final manuscript.
